# Aspects of colorectal cancer screening, methods, age and gender

**DOI:** 10.1111/joim.13171

**Published:** 2020-09-14

**Authors:** R. Hultcrantz

**Affiliations:** ^1^ From the Department of Medicine, Solna Karolinska Institutet Stockholm Sweden

**Keywords:** colonoscopy, colorectal cancer, F‐Hb, screening

## Abstract

Colorectal cancer (CRC) is, besides breast, prostate, lung and skin cancers, the most common cancer worldwide and is suitable for screening. The incidence of CRC varies considerably in different parts of the world: in well‐developed countries, the incidence is between 30 and 70 per 100 000 inhabitants, whereas in less‐developed countries such as sub‐Saharan Africa, it is 10–20/100 000 inhabitants. Women have a lower incidence of CRC, which is usually one‐third of total incidence. Several studies have shown that it is possible to decrease mortality from CRC with about 20%, which is evidenced through the data from countries with screening programmes. Though the method of choice to identify blood samples in faecal matter is under debate, the most feasible way is to perform colonoscopy. Other methods include more advanced faecal analyses, testing for mutations from CRC, sigmoidoscopy, CT colonoscopy or optical colonoscopy. Colonoscopy is in most countries not available in sufficient amount and has to be carried out with great accuracy; otherwise, lesions will be missed to identify, thus leading to complications. Gender is an issue in CRC screening, as women have about 20% fewer colorectal adenomas and CRCs, but they also have more right‐sided lesions, which are more difficult to detect with tests for faecal blood since they create less blood in faeces. Thus, other strategies may have to be developed for women in order for screening to have the same effect. It is essential to introduce colorectal cancer screening in all countries together with other clinical pieces of advice such as information on smoking, obesity and exercise in order to reduce one of the most dangerous cancers.

## Graphical abstract

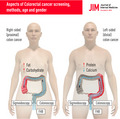

## Introduction

**Fig. 1:**
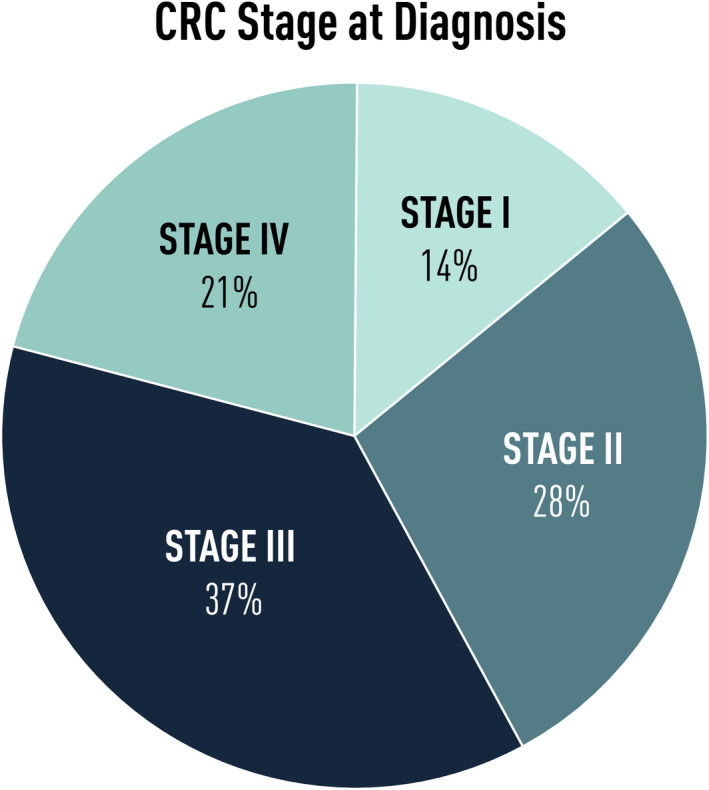
Distribution of the different stages for CRC at the time of diagnosis.

Colorectal cancer (CRC) is, besides breast, prostate, lung and skin cancers, the most common cancer worldwide [1, 2], and the annual incidence of CRC over the world is slightly more than 1 million in men and 79 500 in women, with a mortality rate of 475 000 in men and 387 000 in women. Colorectal cancer can be diagnosed in different from stages I to IV [3], and in developed countries, there is about the same frequency in all stages at diagnoses; thus, 25–30% of CRCs are diagnosed in stage IV, in which there are distant metastases; there is currently no real cure, and in stage I, basically all patients are cured (Fig. 1). Thus, it is thus of utmost importance that the cancer is detected in an early stage. Colorectal cancers occur in all parts of the colon, although most CRCs are localized in the distal part, and 50% are localized in the sigmoid and rectum [4].

In spite of the continuous improved treatment, about 40% of patients die from the disease. Thus, there is still big room for improvement in the management of the disease, and this involves preventive strategies, early detection and treatment. In this context, screening for colorectal cancer has become an important tool.

## Colorectal Cancer (CRC)

### Aetiology, risk factors and prevention

**Fig. 2:**
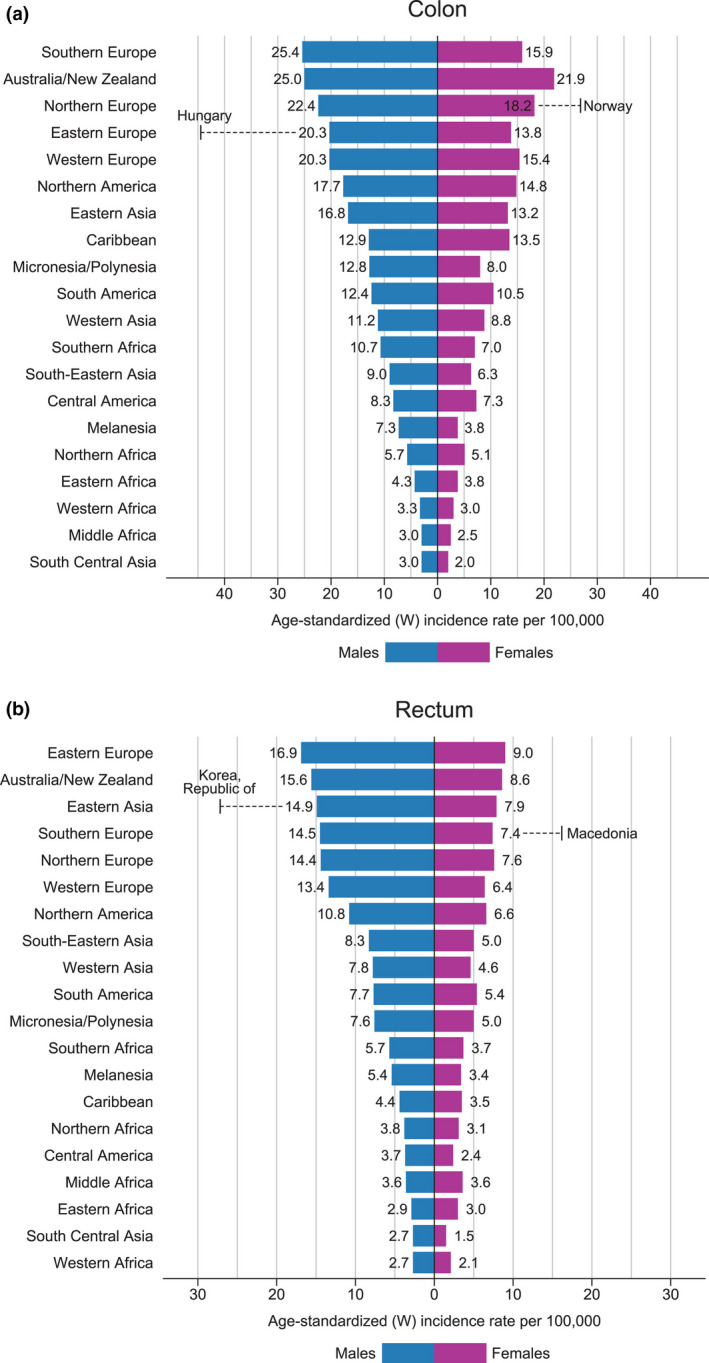
The incidence of CRC in different parts of the world (from WHO *GLOBOCAN* https://gco.iarc.fr/).

The major part of the cancer is sporadic; thus, no major hereditary genetic cause can be found, although chromosomal instability (CIN) is detected in a vast number of sporadic CRC [5]. A clear genetic trait can only be seen in less than five per cent (Fig. 2) and a large number of new insights in this field after the genetic recognition of mutations in the APC gene in patients with familial adenomatous polyposis in 1991 [6], followed by the discovery of the microsatellite instability pathway and the CPG island methylation (CIMP) pathway, seen in Lynch syndrome [7].

Diet has been shown to be of major importance for the incidence of colorectal cancer, and on the bad side, processed meat has been shown to increase the risk for CRC [8]. In environments where the fibre content in the diet is high, there is a lower incidence of colorectal cancer. This was first described by Burkitt in 1955, studying the incidence of colorectal cancer in sub‐Saharan Africa [9]. An important observation has also been in Japanese immigrants to the United States who changed their diet to the one poorer in fibres [10]. In recent years, it has been shown that fibres will affect the number of species in the gut microbiome [11]. The gut microbiome is also likely to be involved in the progression of adenomas to cancer [12]. First, the relative abundance of *Fusobacterium nucleatum* spp. was significantly (*P* < 0.005) elevated continuously from intramucosal carcinoma to more advanced stages. Secondly, *Atopobium parvulum* and *Actinomyces odontolyticus*, which co‐occurred in intramucosal carcinomas were significantly (*P* < 0.005) increased only in multiple polypoid adenomas and/or intramucosal carcinomas [13]. It is not yet proven whether increasing fibres in the diet will alter the occurrence of these species in the gut.

It had been shown that obesity is a risk factor [14], and in a recently published study from the Nurses' Health Study, Nurses' Health Study 2 and Health Professionals Follow‐up Study (45 351 men and 178 016 women, followed for a median of 23 years) on 24 risk factors in relation to risk of cancer in the caecum, ascending colon, transverse colon, descending colon, sigmoid colon, recto‐sigmoid junction and rectum, there were associations between cancers in the proximal parts and hereditary cancers and also female gender. Distal cancers were more related to risk factors such as diet (processed meat), smoking, alcohol and weight [15].

Thus, there are a number of identified risk factors but fewer preventive measures. As is stated above, diet is very important, and increasing evidence shows that fibres can alter the microbiota in the colon leading to a lesser risk for CRC [11]. Although the mechanisms are not altogether clear, it has been shown both in epidemiological studies and in controlled trials that the use of aspirin and other nonsteroidal anti‐inflammatory drugs leads to a decreased risk for CRC [16, 17, 18], and that clinical trials have shown a decreased risk for adenomas in patients with Lynch syndrome if they are treated with aspirin [19].

### Incidence and trends of colorectal cancer

The incidence varies considerably worldwide even between neighbouring regions (Table 1 and Fig. 2) [20]. Countries such as Norway, Denmark, Hungary and South Korea have a very high incidence of over 45/100 000 in men and 37/1 000 000 in women, whereas some countries such as Sweden have a lower incidence of 31/100 000 in men and 25/100 000 in women. Thus, the incidence can differ considerably even in neighbouring countries. The cause for these big differences between neighbouring countries such as Sweden and Norway and Czechia and Slovakia (Table 1) is not clear. There is no reason to believe that differences in registration or other administrative matters are the origin of the variations. However, these variations will of course lead to some countries having to be more prone to initiate screening and other preventive measures.

**Table 1:** Age‐standardized incidence and mortality rates of colorectal cancer in men and women by world regions and countries. The table shows that neighbouring countries may have big differences in incidence/100 000 and mortality (ASR world)

Country	Incidence		Mortality	
	Men	Women	Men	Women
Australia and New Zealand	42	32	12	8
Africa	8	7	5	5
South Africa	17	14	10	5
Middle Africa	7	7	5	5
Asia	20	14	10	6
South Korea	59	31	12	6
China	27	19	11	9
India	5	3	4	3
Thailand	18	14	10	7
Japan	49	29	15	9
Latin America	17	15	9	7
North America	29	21	10	7
United States	28	21	9	8
Canada	34	27	12	8
Northern Europe	37	26	13	9
Finland	28	21	11	7
Norway	46	38	15	11
Denmark	45	36	14	10
Sweden	31	25	12	9
Germany	30	20	13	7
Western Europe	24	22	13	8
United Kingdom	36	25	13	9
Ireland	42	26	15	10
Central and Eastern Europe	37	23	20	12
Hungary	70	38	31	15
Slovakia	70	31	29	15
Czech Republic	42	24	17	9
Austria	26	15	12	6
Switzerland	27	18	10	6
France	36	22	13	8
Southern Europe	40	23	15	8
Spain	44	23	17	8
Italy	25	23	13	8
Greece	31	24	12	7

Data shown are quoted from World Health Organization (WHO), International Agency for Research on Cancer (IARC) https://gco.iarc.fr/.

Colorectal cancer usually appears after 50 years of age, but in recent years, an increase in CRC has been found in younger age groups, especially in Europe [21] and in the United States [22]. In many other countries, there has also been a slight age‐standardized increase in colorectal cancer in younger ages [23].

Colorectal cancer is more common in men than in women, and colorectal adenomas are also more common in men than in women. Women also more often have proximal localization of CRC than men [4].

## Colorectal adenomas

Adenomas are present in up to 20% of persons over 50 years of age, in many cases, up to 50%. Most of the colorectal cancers will develop from adenomas by the adenoma carcinoma pathway, which was described in 1990 by Fearon and Vogelstein [24], although this process has subsequently been shown to be much more complicated [25].

Adenomas develop from hyperplastic epithelium and contain initially low‐grade dysplasia, which develops into high‐grade dysplasia and subsequently invasive carcinoma, a process that may take many years. It is not completely clear how often they develop into cancer, but it has been estimated that during a 10‐year period, 5% will become malignant [26].

There are two types of adenomas in the colon. In the adenomatous pathway to cancer process, it is possible to find different types of mutations in the epithelium in both tumour suppressor genes such as APC and p53 and oncogenes such as KRAS, which can be found in faecal samples from patients with adenomas and cancers. This has been used in the development of faecal screening tests [27].

In the last decade, another type of adenoma has been identified called sessile serrated lesions, which many times do not show dysplasia, but are still regarded as precancerous lesions [28]. Sessile *s*errated polyps* *include hyperplastic polyps (HPs), sessile serrated lesions (SSLs) and traditional serrated adenomas (TSAs). The sessile serrated pathway leading to cancer involves KRAS, p53 and APC mutations (TSA) or BRAF, MLH, p53 and APC mutations. Hyperplastic polyps are the most common (75%) of all serrated polyps. SSLs are approximately 25% of serrated polyps (Fig. 2). SSLs are usually larger, located in the proximal colon, and their endoscopic appearance differs from HPs. TSAs are the least common type of serrated polyps and are typically polypoid lesions found in the distal colorectum. SSLs and TSAs are each considered precursor lesions for CRC. Since many are located in the right colon and are less likely to bleed, they will not be detected in a screening programme based on FOBT or sigmoidoscopy [27, 29]. However, they are important to detect, since patients with detected serrated lesions have an increased risk for CRC after more than three years [30].

## Screening for colorectal cancer

Screening for CRC is now accepted in most countries with organized health care as a way to reduce mortality from CRC [4]. Screening of disorders is carried out according to some rules, which were consented to many years ago: it should be a common disorder; it should be possible to treat; treatment should reduce mortality; it should be easy to diagnose; and it should be cost‐effective to treat. All these rules are achievable in colorectal cancer, and it is accepted in all major countries that screening should be carried out for colorectal cancer [4].

## Methods for colorectal cancer screening

### Faecal occult haemoglobin testing (FOBT)

Adenomas and carcinomas in the colon will bleed, leaving traces of haemoglobin in the faeces, which can be identified with faecal haemoglobin measurements (F‐Hb), and this has been the basis for the first screening trials [31, 32, 33, 34].

Measuring FOBT with the guaiac method (gFOBT) was the first method used to show efficiency in CRC screening. Both adenomatous polyps and cancers have been shown to bleed intermittently, and persons with a positive F‐Hb have been subjected to colonoscopy. The presence of haem was previously measured with guaiac method which gave a yes/no answer for the presence of haemoglobin not only from human blood but also from other species, and it was typically positive in two per cent of patients in the age range of 50‐75 years analysed in these studies (50‐75 years of age). Since the lesions only bleed intermittently, studies included annual or biannual sampling from 15 to 25 years. This technique was used in the four studies showing a reduction in mortality due to CRC ten years after screening between 15 and 30% depending on which invitation technique was used, and a meta‐analysis of these studies has shown a reduction in mortality of about 16% [4]; no analysis of the incidence of CRC has been presented from these studies.

In the last decade, faecal immunological test (FIT),a quantitative test for the presence of human haemoglobin, faecal immunological test (FIT) has been introduced in screening, which has given much new information about the blood content in faeces from patients with various lesions in the colon. As this test is quantitative, it is possible to use different cut‐off values to select participants who should be subjected to colonoscopy, something that was not possible with the guaiac technique. This has led to new insights into how much blood is present in participants with different types of lesions and differences in the presence of blood in faeces in men and women. We now know that with different cut‐offs for haemoglobin concentration, the sensitivity for CRC is between 75 and 95%, and for advanced adenomas, it is between 60 and 80% [35]. There are still interval cancers reported, which means the technique is not optimal. No study has so far been carried out with FIT showing a reduction in CRC mortality. However, the ongoing COLONPREV, CONFIRM and SCREESCO studies are designed to show such a reduction.

It has been shown that not performing colonoscopy on time following a positive FOBT test is a risk [36].

Although the previous screening trials have been performed with guaiac testing, the most current programmes are now run with FIT [4].

Only FIT‐positive persons are examined with colonoscopy, and thus, there is a possibility that FIT‐negative persons with advanced adenoma or CRC can develop a symptomatic CRC between two samples. This is called interval cancer, and it occurs in FIT programmes [37, 38]; the incidence of interval cancers in FIT screening is much lower than regular incidence. They are more often seen in female patients and more in persons with right‐sided cancer.

### Other faecal test

A problem in most countries other than the United States is a lack of colonoscopy resources and also the fact that colonoscopy is a rather tedious and sometimes dangerous way to examine the colon. This is a reason to increase specificity in the test prior to colonoscopy. A questionnaire has been tested with good results [39], based on age, gender, smoking status, family history, body mass index and self‐reported diabetes, and this increased the specificity for CRC.

Another way is to add tests to FIT to increase the specificity. A test that is used in the United States is developed to identify common mutations in CRC in the faeces [27]; another is a recently developed microRNA technique, which can improve the selection of screening participants who shall be chosen for colonoscopy [40]. Other methods that have been tested are combination of FIT and calprotectin, which also increased specificity to 90%. More work on this can certainly help decrease the number of colonoscopies needed in screening.

### Sigmoidoscopy

Four studies have shown that screening for colorectal cancer with sigmoidoscopy will lead to a decrease in CRC ten years after the examination [41, 42, 43, 44]. The numbers shown indicative of a reduction of 33% in the incidence of CRC and a reduction in mortality of about the same rate. In these four studies, the patients were initially examined with sigmoidoscopy after a preparation with enema, and if polyps were found, a subsequent colonoscopy was performed. The participation in the studies differed considerably from 25% to 65% possibly because of the countries they were conducted in and the difference in the invitational method. A meta‐analysis of these studies showed that 52 adenomas needed to be removed in order to avoid one CRC [45]. This method has been introduced in England from the age of 56 years [46], and in the parts of Italy [47].

### Colonoscopy

**Fig. 3:**
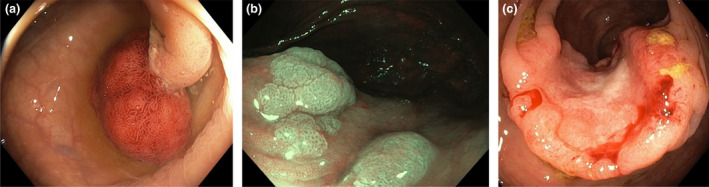
Three different stages in the adenoma carcinoma sequence. (The figures are shown with courtesy of Stefan Willmarsson) (a) Traditional big adenomatous polyp with a prominent stalk. (b) A sessile serrated polyp. (c) Colorectal cancer with ulceration.

Colonoscopy is the gold standard for the examination of the colon, and lesions can be identified through colonoscopy (Fig. 3a‐c). There are no screening studies with long‐time results, but several publications indicate that if a person has undergone a colonoscopy, the risk of developing a CRC after the examination is reduced for more than 10 years [48, 49]. In fact, in the latter of these studies the risk was reduced for up to 15 years [49].

There are four ongoing studies currently designed to show whether colonoscopy is efficient as a method for CRC screening, NORDICC [50], COLONPREV [51], CONFIRM [52] and SCREESCO (*NCT02078804*). The NORDICC study only uses colonoscopy and compares these participants with a control group, whereas the COLONPREV, CONFIRM and SCREESCO also have arms with FIT, which are compared with the control arm. In all types of screening, the rate of participation is of importance, and in colonoscopy studies, the participation seems lower than in screening with FIT, leading to a risk of decreased reduction in mortality. However, during a colonoscopy most cancers and adenomas will be removed from the colon as opposed to the FIT method, which will detect 50–90% of adenomas and cancers.

Although colonoscopy is an excellent tool to detect and to treat lesions in the colon, in screening it has to be carried out with high accuracy, failing which there is a possibility to harm. The risk of perforation and bleeding is well recognized and may also lead to lethal complications [53]. Thus, screening procedures have to be carried out by highly trained colonoscopists, and therefore, systematic training is important and has been shown to be effective [54, 55]. It has also recently been shown from the Polish programme that even if all lesions are removed during a screening colonoscopy, there is a risk for CRC if the lesions are bigger than 20 mm, whereas if they are smaller, there is a decreased risk compared with those with no lesions [56]. In recent years, it has been shown that even though colonoscopy is the gold standard, the risk of postcolonoscopy colorectal cancers (PCCRC) is quite high, up to 10% [57, 58, 59], and often depends on lacking quality in how the procedure is carried out [60].

### CT colonoscopy

**Fig. 4:**
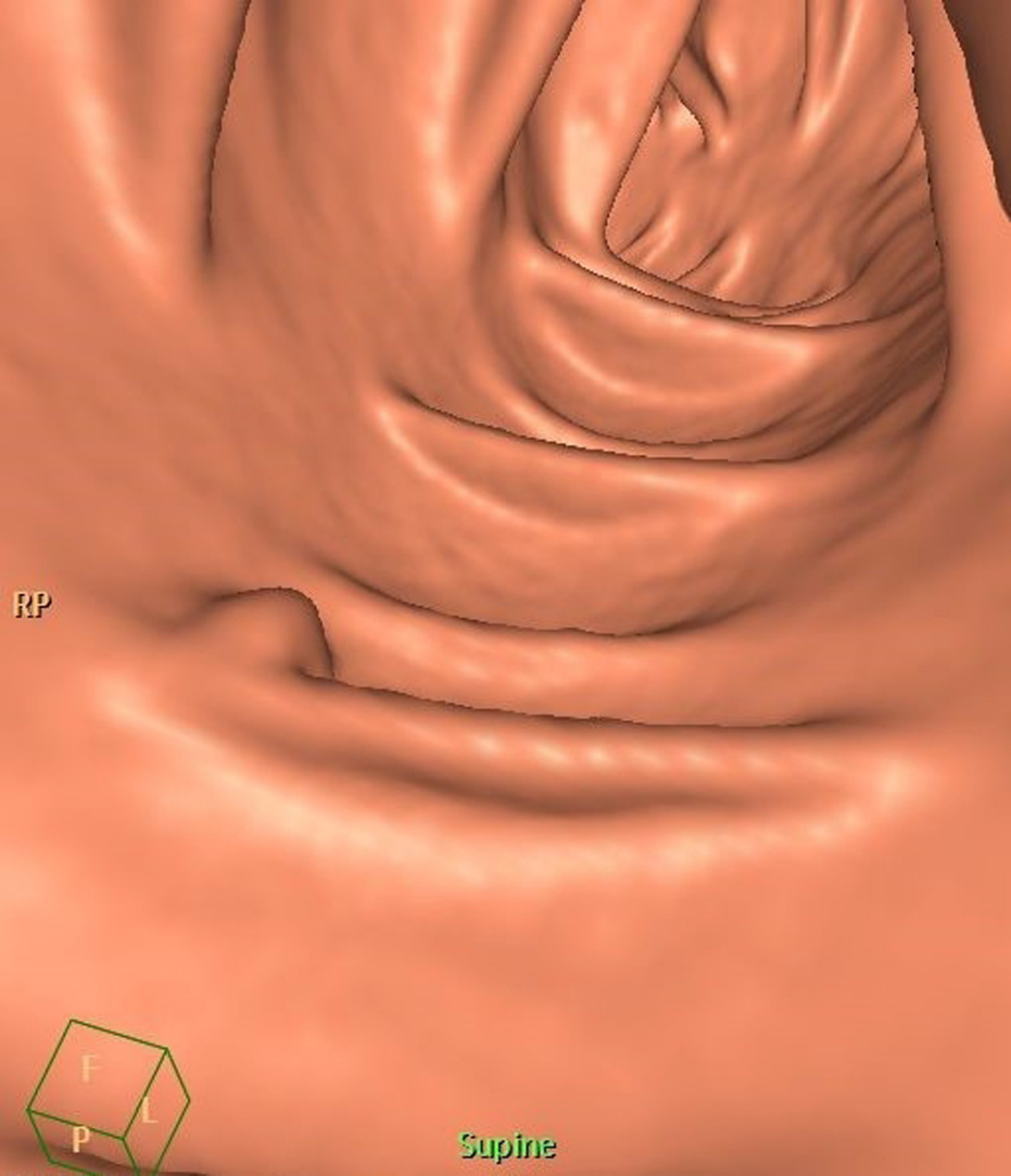
Computerized tomography of a small polyp in a so‐called fly‐through image (Courtesy: Mikael Hellström).

CT colonoscopy technique has developed enormously and is now excellent; however, the major drawback in it is the same as that of colonoscopy, namely that the bowel has to be cleaned with the same methods, a procedure considered to be tiresome and is often unpleasant. Furthermore, the method offers examination, and the images (Fig. 4) can be re‐examined as compared to optical colonoscopy. However, no treatment can be performed. There have been several studies comparing these two methods, and the conclusion is still that optical colonoscopy has many advantages over CT colonoscopy. Thus, this method has not yet been accepted as a primary screening tool in population‐based screening. It is used if colonoscopy cannot be successfully performed or if the participant refuses to undergo examination with colonoscopy. However, there is now also a technique called faecal tagging. A contrast agent is swallowed and tagged to the faeces, and faeces will have a different contrast compared with the mucosa; thus, cleaning of the bowel may not be necessary in future [61, 62], although this is currently not good enough to be used for screening purposes. Furthermore, CT colonoscopy has in many studies been shown to be effective, but not in a large screening study [31, 32, 33, 63, 64].

## The current screening situation worldwide

Most developed countries have some kind of colorectal cancer screening programme [4]. The major difference is whether it is population‐based or opportunistic. In the United States, which has opportunistic screening, the U.S. Preventive Services Task Force (USPSTF) recommends one of the following four methods: FIT and colonoscopy annually from 50 years of age; CT colon every three years; sigmoidoscopy every five years; or colonoscopy every 10 years [65].

In Europe, the methods are mostly similar (FOBT), although the implementation is different. Twenty‐two countries in the European Union have or plan to initiate a strict population‐based programme where everyone above 60 years is invited biannually to FIT testing with a subsequent colonoscopy, whereas others such as Greece and Latvia have opportunistic screening. Germany, Poland, Norway and Sweden have made decisions and are introducing population‐based programmes.

A detailed description of the worldwide practice is given in the IARC Handbooks of Cancer Prevention Volume 17 on Colorectal Cancer Screening (pages 51–79) (ISBN 978‐92‐832‐3022‐9).

## Compliance and participant’ experience in CRC screening

Compliance is of great importance in any kind of screening. There are various national or regional screening programmes with different guidelines. First, it is of great importance if the participant has an insurance, which covers the cost of screening. This is not a big problem in Europe but is indeed a problem in other parts of the world. The method of invitation is of great importance. Furthermore, the method used in the programme varies from FOBT, sigmoidoscopy to colonoscopy. Thus, it is difficult to compare the effectiveness of different programmes for many reasons.

Compliance and ways to increase compliance are mostly studied in areas with F‐Hb screening. In England where CRC screening with guaiac‐based screening has been practised since 2003, the uptake rate was about 60% until the introduction of the FIT instead of the guaiac test, and when it increased to about 70% [66]. A similar effect has been seen in Stockholm, the biggest region in Sweden, where the compliance also increased from about 60 to 70% [67]. Also, in the Netherlands a high compliance is seen [68]. In other countries with FIT such as France [69], Italy [70] and Spain [71], the compliance is as low as 30%. The uptake also varies considerably within the country. Thus, in areas with low socio‐economic status it has been shown that the uptake is lower. Other factors that seem to lower the participation are immigration and bad health status. In many countries in Europe, the participants receive an invitation by mail and the letter can contain the test kit, which probably is a preferred way to do it. In other countries, the participant has to go to a pharmacy to pick up the test kit. A third way is opportunistic screening, meaning that it is completely up to the participant to take the initiative to participate, a method that usually leads to a low participation.

The examination method used is also of importance, and FOBT usually leads to a higher participation rate than in programmes with sigmoidoscopy, where the participation in the sigmoidoscopy arms has been shown to be lower than in the FOBT arms.

The role of the local general practitioner or family doctor has also been examined since decision‐making to participate or not is often based on cultural and environmental influences, and a personal doctor is usually someone the patient relies on. In the English programme which is organized, this was shown to be positive [72], and it has also been shown to be positive in opportunistic programmes in Asia [73]. In screening programmes in which participants receive invitation by mail, the role of the local doctor may be of less importance, as opposed to programmes such as in the United States where a referral for colonoscopy has to be written by a doctor. Individuals invited to the SCREESCO study did not see the relevance of involving any healthcare providers in their decision [74]. Other studies show that a physician recommendation is an important factor for participation [75].

A Systematic Review and Meta‐Study Synthesis [76], from Canada, showed that the awareness of appropriate colorectal cancer screening and the indication for screening were a facilitator for participation. In contrast, a qualitative study based on focus group discussions and individual interviews showed that both participants and nonparticipants invited to the Screening of Swedish Colons (SCREESCO) study lacked knowledge on CRC and screening [74]. Both groups agreed that values in society were the main reason and that it was important to avoid frightening information.

Health literacy is a concept recently introduced and is important for how individuals understand and use information [77]. Some previous studies measuring health literacy in relation to CRC screening participation have, however, shown contrasting results [78, 79, 80]. The results, based on 1498 individuals invited to the SCREESCO from the study (nonparticipants, *n* = 164), were in line with those from the studies showing no difference in health literacy amongst participants and nonparticipants. A majority (about 90%) of the individuals displayed acceptable levels of health literacy independent of group affiliation [81]. However, both groups expressed that, as a complement to the invitation letter, it is important to be able to obtain information on CRC from different sources, such as web pages, leaflets and telephone calls [81]. Psychological distress and anxiety have also previously been suggested to be a barrier to CRC screening and a potential side effect related to being invited to CRC screening [82, 83]. The results from a substudy in SCREESCO showed that a majority (80%) had lower levels of anxiety in relation to their decision to participate or not, but female gender was associated with higher anxiety scores [84]. In a qualitative study, values and preferences were found to be more important for participation in CRC screening [74]; nonparticipants have a more fatalistic approach such as ‘what happens happens’, whereas participants having viewed CRC screening as a way of ‘having control over one’s health' [74]. Moreover, the influence of other persons promoted participation and prevented it amongst nonparticipants. This is in line with previous research showing that social norms play a role for screening behaviour [85]. Work situation also played a role for prioritizing CRC screening, where nonparticipants expressed that being off work was not an option [79].

## Health economy

One of the goals with screening for diseases is that it should ease the burden of disease for health care in the country, and a number of studies have been addressing this [86, 87, 88, 89, 90, 91]. There is convincing evidence from health economy models that this is the case even in a running programme [47].

However, as for the efficacy of CRC screening, when it comes to reduction in mortality it is also difficult to show the effects of colorectal cancer screening programmes. First, there is an increase in the incidence of CRC since there is an active search going on. Secondly, the number of CRC found during a period is only a portion of those detected because of symptoms. As compared to breast cancer screening, CRC screening programmes also include polypectomy, which means that precancerous lesions are ablated, leading to a decrease in the incidence of CRC [41, 42].

## Gender and age differences in CRC screening

**Fig. 5:**
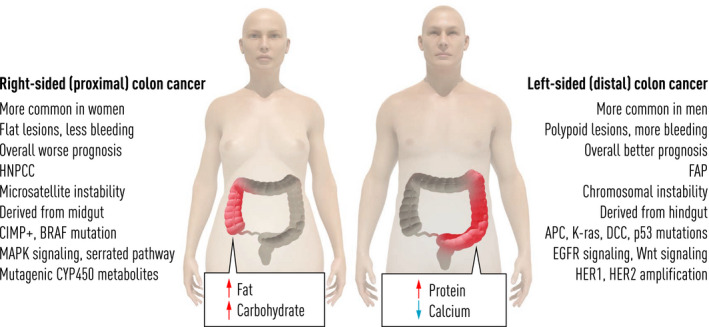
Most important sex differences between men and women. Right‐sided tumours are more common in women and patients with HNPCC and seem to be correlated with high intake of carbohydrates and fat, and they have more microsatellite instability and BRAF mutations. Left‐sided lesions are more common in men and patients with FAP, and they are correlated with high intake of protein (meat) and calcium and have more APC, KRAS and P53 mutations.

Women have lower incidence of CRCs, and colonoscopy studies show that they have fewer colorectal adenomas than men (Fig. 5). Women have more right‐sided cancers, which are more difficult to find in screening [92, 93], and these cancers also show more microsatellite instability, typical of right‐sided cancers [94]. Bleeding in colon lesions differs between men and women [35], and it is a well‐known fact that women have less haemoglobin in faeces than men [95, 96, 97]. In studies where the same cut‐off is used for men and women, there is evidence that screening in women is not as efficient; thus, one cannot show a reduced mortality. It has been suggested that the cut‐off level for FIT should be lower for women than men in order to find as many CRC in both genders [98]. Are the fewer colorectal adenomas and carcinomas correlated with the lower levels of haemoglobin in faeces or are other mechanisms involved? The ultimate question is of course whether there should be different cut‐off levels for men and women in FIT screening. A few studies have been addressing this question. In a substudy to the SCREESCO study in which all participants undergoing colonoscopy also tested for FIT, we could find that the level of FIT was correlated with the findings of adenoma and carcinoma [96, 99]. Other studies have supported this finding [97, 100, 101], and thus, the effect on screening results of different cut‐off levels for men and women is not yet clarified. In fact, in the Netherlands, it was shown that the current model was recommended based on a study comparing findings in men and women [102].

In one of the studies on sigmoidoscopy, the NORCCAPP study, the findings indicate that there was no real reduced mortality in the female groups [43].

## Changes in mortality from CRC following introduction of colorectal cancer screening

It is difficult to show the effects of screening programmes outside clinical trials [103]. The difference between trials and regular screening programmes is termed effectiveness versus efficacy. It has been shown that there is a decrease in the incidence of CRC in areas where screening is ongoing [104, 105, 106]. However, there are also areas such as in Finland, where there is no evidence of an effect of the ongoing screening programme [107]. In the United States, there is a reduction in the mortality from CRC in many areas in persons above 60 years [22], and an increase in the age groups not subjected to screening. In the United States where we find FOBT screening and to a larger extent colonoscopy screening, it is estimated that about 80% of the population above 60 years has undergone a colonoscopy. However, it can also be noted that CRC incidence was decreasing in countries before screening was organized, such as Denmark, Sweden and Switzerland [20].

## Negative effects of screening

Colorectal cancer screening has obvious good effects on population health and the economy. Screening has however some negative effects, and there are professional groups that think screening should be abolished. A major concern is often that in some of the published studies, the total mortality was not altered, even though mortality from CRC was lower. However, in a disease that is not one of the big causes of death, this cannot be expected. Furthermore, CRC screening may in many countries demand colonoscopy resources, which are hard to obtain, since these resources are scarce in many countries. Yet, one objection is also that poor skills in colonoscopy performance may lead to complications with even hospitalization and death; however, with training and follow‐up in registries this may be kept at a very low level and underachieving colonoscopists can also be taken out of screening procedures. Finally, as has been described above, participants may be disturbed due to reminders of a dangerous disease, but this seems to be smaller problem.

## Future prospects of CRC screening

Although it is now more than 20 years ever since the results from the four big FOBT studies were published, population‐based screening is not introduced in all industrialized countries, not even in those with functioning organized health care set‐up. Thus, the priority should be to focus on introducing some kind of screening programme, since CRC is such an important factor in cancer mortality. It has been shown that both FOBT screening and screening with sigmoidoscopy decrease mortality from CRC. FOBT screening is comparably easy and not too expensive to introduce in a country with good infrastructure and health care. As described above, it relies on many factors in order to achieve a high participation, such as that health care is equally available for the whole population, the invitation method, the choice of method, that health care can provide examinations and that the population is well informed.

As has been described above, in Western countries there has been a decrease in CRC incidence in older age groups and an increase in younger age groups. This means that we have to reconsider which age groups should be invited. Since the incidence has had a peak at 75 years of age, the studies have included age groups 50–75 or 60–75. It has been shown that screening over the age of 70 years with colonoscopy in an American population did not lead to increased survival [108]. In view of the lower incidence in older age groups and increase amongst younger age groups which has been observed in the last years, one should probably reconsider whether the current recommendations are correct [20, 21, 22]. As is shown in Table 2, there are now four big trials including colonoscopy and FIT [50, 51, 52] and the Swedish study SCREESCO (*NCT02078804*), which will lead to much information on the value of CRC screening. It is unfortunate that none of the trials include persons younger than 50 years of age.

**Table 2:** Summary of the ongoing randomized trials in colorectal cancer screening with mortality from and incidence of CRC as end‐points

Study	Country	Method	Inclusion start year	End‐points	Participants number	Age years	Completion date year
NORDICC^a^	Norway	Colonoscopy	2009	Mortality from CRC	24 000	55–64	2036
	Sweden	Controls		Incidence of CRC	44 000		
	Poland						
	Netherlands						
COLONPREV^b^	Spain	Colonoscopy	2009	Mortality from CRC	27 749	50–69	2021
		FIT		Incidence of CRC	27 749		
CONFIRM^c^	USA	Colonoscopy	2012	Mortality from CRC	25 000	50–75	2028
		FIT			25 000		
SCREESCO^d^	Sweden	Colonoscopy	2014	Mortality from CRC	30 500	60	2034
		FIT		Incidence of CRC	60 000		
		Controls			183 000		

^a^ Bretthauer et al. ref [50].

^b^ Quintero et al. ref [51].

^c^ Dominitz et al. ref [52].

^d^ SCREESCO Clin trials. Gov *NCT02078804*.

Furthermore, the ideal way forward may be to offer persons several options based on what they prefer: FIT, CT colonoscopy, sigmoidoscopy or optical colonoscopy. It is also possible to use not only FIT but also questionnaires to find persons with higher risk for CRC to optimize the use of colonoscopy.

## Conclusions

Studies have shown that screening for CRC with several methods leads to reduction in CRC mortality, and also, in countries with screening there is evidence that the incidence of CRC is decreasing. Screening should be population‐based, and all citizens should be offered screening. The method of choice is an initial test for the presence of blood in faeces, and in those with blood, a high‐quality colonoscopy is performed. The amount of blood can be monitored, and different cut‐offs can be used, often depending on how many colonoscopies health care can perform. Women have less blood in faeces but also fewer colonic lesions. Sigmoidoscopy is also shown to be efficient but less efficient for women. Colonoscopy trials are ongoing, but none is finished. The future of screening will include new methods with a better pre‐evaluation before colonoscopy and more gender‐equal methods.

## Conflict of interest statement

The author declares they have no conflict of interest.
